# Glutaredoxins employ parallel monothiol–dithiol mechanisms to catalyze thiol–disulfide exchanges with protein disulfides†
†Electronic supplementary information (ESI) available: Determination of reduction potentials; discussion of Scheme 2b and literature examples of dithiol mechanism for EcGrx1; Tables S1 and S2; Fig. S1–S4. See DOI: 10.1039/c7sc04416j


**DOI:** 10.1039/c7sc04416j

**Published:** 2017-12-06

**Authors:** Ashwinie A. Ukuwela, Ashley I. Bush, Anthony G. Wedd, Zhiguang Xiao

**Affiliations:** a School of Chemistry , Bio21 Molecular Science and Biotechnology Institute , The University of Melbourne , Parkville , Victoria 3010 , Australia; b Melbourne Dementia Research Centre , Florey Institute of Neuroscience and Mental Health , The University of Melbourne , Parkville , Victoria 3052 , Australia . Email: zhiguang.xiao@florey.edu.au

## Abstract

Glutaredoxins (Grxs) are a family of glutathione (GSH)-dependent thiol–disulfide oxidoreductases. They feature GSH-binding sites that directly connect the reversible redox chemistry of protein thiols to the abundant cellular nonprotein thiol pool GSSG/GSH. This work studied the pathways for oxidation of protein dithiols P(SH)_2_ and reduction of protein disulfides P(SS) catalyzed by *Homo sapiens* HsGrx1 and *Escherichia coli* EcGrx1. The metal-binding domain HMA4n(SH)_2_ was chosen as substrate as it contains a solvent-exposed CysCys motif. Quenching of the reactions with excess iodoacetamide followed by protein speciation analysis *via* ESI-MS allowed interception and characterization of both substrate and enzyme intermediates. The enzymes shuttle between three catalytically-competent forms (Grx(SH)(S^–^), Grx(SH)(SSG) and Grx(SS)) and employ conserved parallel monothiol and dithiol mechanisms. Experiments with dithiol and monothiol versions of both Grx enzymes demonstrate which monothiol (plus GSSG or GSH) or dithiol pathways dominate a specific oxidation or reduction reaction. Grxs are shown to be a class of versatile enzymes with diverse catalytic functions that are driven by specific interactions with GSSG/GSH.

## Abstract 2

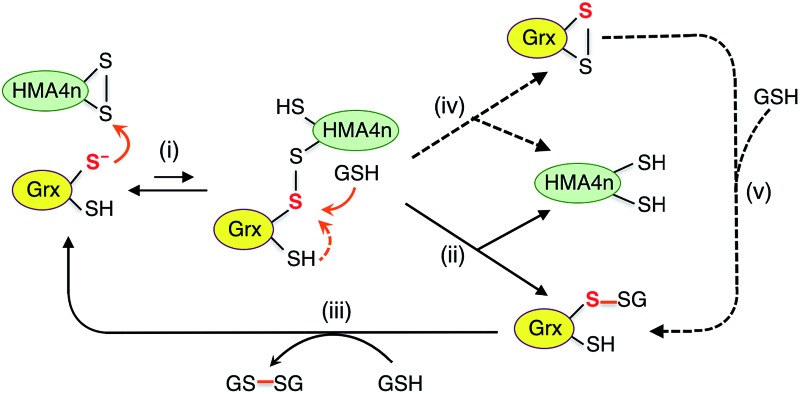
Glutaredoxins were demonstrated to be a family of versatile enzymes capable of catalyzing thiol–disulfide exchange involving GSSG/GSH *via* different catalytic routes either alone or in parallel.

## Introduction

**Fig. 1:**
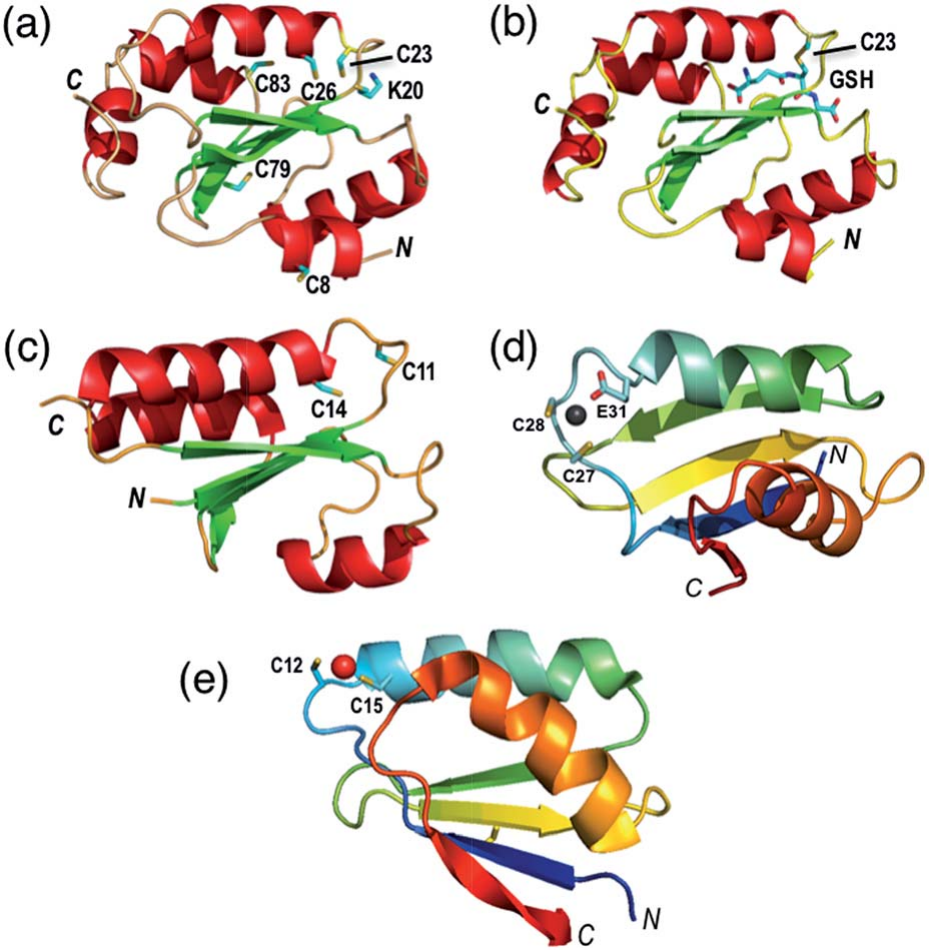
Protein molecular structures. (a) Fully reduced HsGrx1 (PDB: 1JHB; thioredoxin fold); (b) HsGrx1(C8,26,79,83S)–GSH complex (; 1B4Q); (c) reduced EcGrx1 (; 1EGR); (d) Zn(ii)-AtHMA4n (; 2KKH; ferredoxin fold); (e) Cu(i)-Atox1 (; 1TL4). Labelled amino acid residues and the GSH fragment are shown as sticks while the metal ions in Zn(ii)-AtHMA4n and Cu(i)-Atox1 are represented as spheres.

The thioredoxin family of enzymes catalyze biological thiol–disulfide exchange reactions and play vital roles in a wide spectrum of cellular functions including redox sensing, cell signaling, cellular redox homeostasis, oxidative protein folding, regulation of protein thiol function and apotopsis.^1^–^7^ They share a common thioredoxin fold featuring a conserved Cys–xx–Cys active site (Fig. 1a–c).^8^–^10^ The Cys residue closer to the N-terminus is solvent-exposed with a low p*K*_a_ (<5 in most cases) that allows its thiolate anion to launch nucleophilic attack on a target disulfide bond.^6^,^11^,^12^

The family includes thioredoxins (Trxs), glutaredoxins (Grxs), protein disulfide-isomerases (PDIs) and the disulfide bond protein family A–D (DsbA–D).^6^,^7^ The Grxs are unique in being dependent for their catalytic activity upon the abundant cellular GSSG/2GSH redox couple where GSH is glutathione.^6^,^13^–^15^ During catalysis, they shuttle between three competent oxidation states, namely, the dithiol Grx(SH)(S^–^), the internal disulfide Grx(SS) and the glutathionylated form Grx(SH)(SSG).^16^

There is at least one GSH-specific binding site that enables the Grxs to act as scaffold proteins for the assembly and delivery of GSH.^17^ X-ray crystal structures are available in which the GSH molecule interacts with a group of highly conserved residues and forms Grx(SH)(SSG) *via* the reactive N-terminal Cys (Fig. 1b).^18^–^20^

We have recently re-evaluated the reduction potentials of two Grx enzymes, *H. sapiens* HsGrx1 and *E. coli* EcGrx1 (Fig. 1a and c).^16^ They exhibited similar standard reduction potentials *E*o′P(SS) ∼ –170 mV (*vs.* SHE) at pH 7.0. This is significantly more positive than those (∼–270 mV)^24^,^25^ reported for Trxs, but more negative than those (∼–125 mV)^16^,^26^,^27^ of DsbAs. Trxs are generally believed to function as disulfide reductases while DsbAs are known as protein dithiol oxidases. The intermediate reduction potentials of Grxs mean that they may function as either reductases or oxidases, depending upon the cellular conditions imposed by the GSSG/2GSH redox couple. Notably, the reduction potential for both catalytically competent oxidized forms Grx(SS) and Grx(SH)(SSG) were demonstrated to vary with GSH concentrations.^16^

The apparent half-cell reduction potential defined by the GSSG/2GSH redox couple correlates with the biological status of the cell: ∼–240 mV for proliferation, ∼–200 mV for differentiation and ∼–170 mV for apoptosis.^28^ Importantly, relative concentrations of GSSG/GSH vary considerably across different cellular compartments and so do the formal reduction potentials imposed by the GSSG/2GSH couple.^29^ Consequently, the catalytic functions of Grxs are linked directly to the redox state of GSSG/2GSH *via* the Nernst equation.

**Scheme 1:**
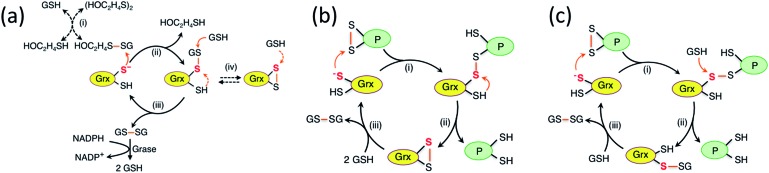
(a) Proposed chemical pre-equilibrium monothiol mechanism for the classic bis(2-hydroxyethyl)disulfide (HEDS) assay.^21^,^22^ It consists of two coupled enzyme reactions employing HEDS as the disulfide substrate; (b and c) proposed dithiol mechanism (b)^15^ and monothiol mechanism (c)^23^ for catalytic reduction of protein disulfide P(SS) by Grx/GSH.

While study of the catalytic oxidation of protein dithiols by Grx/GSSG is limited,^23^ the catalytic reduction of protein disulfides by Grx/GSH has been widely examined and three different mechanisms have been proposed (Scheme 1a–c).^6^,^15^,^21^–^23^,^30^–^33^ These variously involve chemical pre-equilibria and/or monothiol or dithiol mechanisms, depending on the nature of the substrate and Grx enzyme.

To resolve this uncertain situation, the present work undertook a systematic study of the two representative enzymes *H. sapiens* HsGrx1 and *E. coli* EcGrx1 in the catalytic oxidation/reduction of a protein dithiol/disulfide with GSSG/GSH as the electron acceptor/donor system. Quenching of the reactions with excess iodoacetamide (IAA),^12^ followed by protein speciation analysis with electrospray ionization mass spectrometry (ESI-MS), allowed interception and characterization of both substrate and enzyme intermediates and provided new insights into the catalytic mechanisms.

Selected variants of the enzymes were employed to demonstrate that Grxs may employ either a monothiol mechanism or a dithiol mechanism or, more frequently, both in parallel. Consequently, Grxs are defined as a class of dynamic thiol–disulfide oxidoreductases. Their specific interaction with GSH means that their reduction potentials are dependent upon GSH concentration and so their catalytically-competent glutathionylated forms allow versatile catalytic functions.

## Results

### Glutaredoxin enzymes and protein thiol/disulfide substrates

HsGrx1 contains a total of five Cys residues with two present in the active site motif Cys^23^–xx–Cys^26^ whereas EcGrx1 contains only the two that are located in the active site Cys^12^–xx–Cys^14^ (Fig. 1a and c). Protein variants generated included two single mutants HsGrx1-C23S and EcGrx1-C14S (in which the C-terminal Cys of the active site is replaced by Ser), a triple mutant HsGrx1-tm (HsGrx1-C8,79,83S where three non-active site Cys residues are replaced by Ser) and a quadruple mutant HsGrx1-qm (HsGrx1-C8,26,79,83S where, in addition, the C-terminal Cys is replaced by Ser). Production and characterization of these proteins was reported previously.^16^,^23^

Two well-characterized proteins were chosen as dithiol substrates. HMA4n is the N-terminal metal binding domain of the heavy metal transporting P_1B_-type ATPase HMA4 from the plant *Arabidopsis thaliana*. It contains a double CysCys motif in a solvent-exposed Zn(ii)-binding sequence (Cys^27^–Cys^28^–xx–Glu^31^) (Fig. 1d).^34^ It was selected as the primary protein substrate since the two Cys thiols can be oxidized readily to a disulfide. The sulfur redox chemistry of copper metallo-chaperone Atox1 has been studied recently.^23^ It features a high affinity Cu(i) binding motif (Cys^12^–Gly–Gly–Cys^15^) and was used for comparative studies in selected cases. Both HMA4n and Atox1 were oxidized quantitatively by a slight excess of [Fe^III^(CN)_6_]^3–^ to cleanly yield internal disulfides HMA4n(SS) and Atox1(SS). The identities of the reduced and the oxidized forms were confirmed by quantitative thiol assay and ESI-MS analysis. The reduction potentials of Atox1(SS) and HMA4n(SS) are essentially identical at *E*o′P(SS) = –192 mV at pH 7.0 (see ESI, Fig. S1, Tables S1 and S2†).

### Oxidation of a protein dithiol by GSSG

**Fig. 2:**
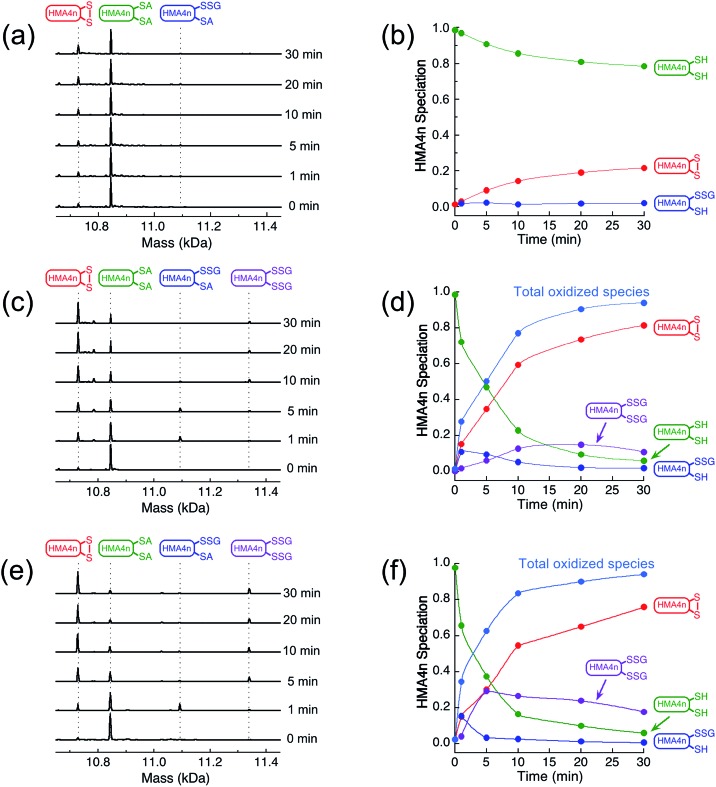
Protein speciation and reaction progress analysis upon oxidation of HMA4n(SH)_2_ (10 μM) in deoxygenated KPi buffer (50 mM, pH 7.0) containing GSSG (400 μM)/GSH (40 μM): IAA/ESI-MS analysis and speciation distribution for non-catalytic oxidation (a and b) and for catalysis by HsGrx1-tm (100 nM; c and d) and by HsGrx1-qm (50 nM; e and f). RS-A refers to the alkylated thiol RS-CH_2_CONH_2_.

The two adjacent cysteinyl thiols in HMA4n(SH)_2_ can be oxidized by GSSG to generate the internal disulfide HMA4n(SS). This reaction is slow (Fig. 2a and b) but is catalyzed by Grx enzymes according to the overall reaction of eqn (1):1
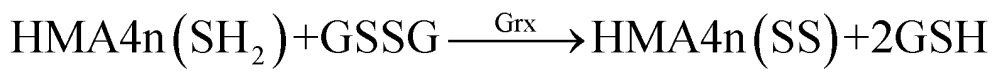



The reaction in KPi buffer (50 mM, pH 7.0) was evaluated by quenching with excess IAA, followed by protein speciation analysis with ESI-MS. Alkylation of protein thiols by IAA is much faster than thiol–disulfide exchange reactions.^16^ Addition of group A = CH_2_CONH_2_ to each accessible free protein thiol P(SH) leads to a net increase in molar mass of 57 Da in P(SA). Reaction (1) was followed under a variety of conditions (Fig. 2).

IAA/ESI-MS analysis confirmed that HMA4n(SS) was the dominant product that increased with reaction time under all experimental conditions (Fig. 2a–f). Two other components, HMA4n(SH)(SSG) and HMA4n(SSG)_2_, were detected at low levels during catalysis (Fig. 2c–f), but not for the non-catalytic control (Fig. 2a and b). When the variant HsGrx1-tm (0.1 μM; containing the active site motif Cys^23^–xx–Cys^26^ as the only Cys residues) was used as the catalyst for oxidation of HMA4n(SH)_2_ (10 μM) in buffer containing GSSG (400 μM)/GSH (40 μM),^35^ the concentration of HMA4n(SH)(SSG) increased quickly and reached a maximum within the first minute to ∼10% of total HMA4n fractions (Fig. 2c and d). Its concentration then decreased steadily while that of HMA4n(SSG)_2_ increased slowly up to ∼15% and then deceased after >80% of HMA4n(SH)_2_ had been oxidized.

**Fig. 3:**
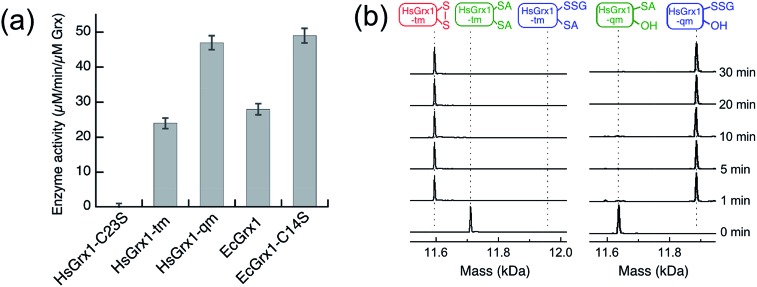
(a) Comparison of substrate turnover rates for catalytic oxidation of HMA4n(SH)_2_ by different Grx enzymes under the condition of Fig. 2; (b) IAA/ESI-MS analysis for HsGrx1-tm (left panel) and HsGrx1-qm (right panel) during the catalysis of Fig. 2. RS-A refers to the alkylated thiol RS-CH_2_CONH_2_.

Unexpectedly, the activity of monothiol HsGrx1-qm (the reactive N-terminal Cys23 is the only Cys present) is about double that of dithiol HsGrx1-tm (Fig. 2c–f and 3a). The appearance and decay of HMA4n(SH)(SSG) and HMA4n(SSG)_2_ are qualitatively similar in each system, but the latter is more abundant with HsGrx1-qm as catalyst (compare Fig. 2c, d and e, f for equivalent substrate oxidation rates). These properties were shared by the equivalent *E. coli* enzymes EcGrx1 and EcGrx1-C14S (Fig. S2†).

IAA/ESI-MS analysis may be applied to the enzymes as well. Only HsGrx1-tm(SS) and HsGrx1-qm(SSG) were detected throughout the catalysis although both were added as the fully reduced forms (Fig. 3b). This indicates that, under the oxidative conditions defined by the GSSG (400 μM)/2GSH (40 μM) redox couple, these oxidized Grx forms acted as the resting enzymes ensuring their optimal oxidase function.

The overall oxidation rates are defined most adequately by the consumption rate of substrate HMA4n(SH)_2_. Variation of enzyme concentration allowed estimation of the turnover rate (Fig. 3a). HsGrx1-tm and EcGrx1 exhibit comparable catalytic activities with an average substrate turnover rate of ∼26 μM per min per μM per enzyme (Table 1). They each feature an intact Cys–xx–Cys active site (Fig. 1a and c). Mutation of the N-terminal reactive Cys^23^ to Ser in HsGrx1 abolished the enzyme function completely while mutation of the C-terminal Cys to Ser in both HsGrx1-tm and EcGrx1 effectively doubled the enzyme activity (Fig. 3a, Table 1).

**Table 1:** HMA4n turnover rates by Grx enzymes^*a*^

Enzyme	As an oxidase^*b*^ (μM per min per μM per enzyme)	As a reductase^*c*^ (μM per min per μM per enzyme)
HsGrx1-C23S	∼0	0.2(1)
HsGx1-tm	24(1)	5.9(5)
HsGrx1-qm	47(2)	6.9(6)
EcGrx1	28(2)	6.1(5)
EcGrx1-C14S	49(2)	7.1(5)

^*a*^The background substrate turnover rate under the same conditions without enzyme was subtracted in each case.

^*b*^In KPi buffer (50 mM, pH 7.0) containing GSSG (400 μM)/GSH (40 μM), HMA4n(SH)_2_ (10 μM) and a Grx enzyme (0–20 nM) (see Fig. 2); the bracketed values are the errors in the last digits averaged over three measurements.

^*c*^In KPi buffer (50 mM, pH 7.0) containing GSSG (20 μM)/GSH (800 μM), HMA4n(SS) (10 μM) and a Grx enzyme; the bracketed values are the errors in the last digits estimated from a linear fitting of the reduction rates with [Grx] in the range of 0–100 nM (see Fig. 4d).

The above observations lead to several conclusions: (1) HMA4n(SH)(SSG) is a key intermediate species on the pathway to the final oxidation product HMA4n(SS) but may be trapped partially as the doubly glutathionylated form HMA4n(SSG)_2_ also, especially when the monothiols HsGrx1-qm or EcGrx1-C14S were used as catalysts (Fig. 2c–f); (2) HMA4n(SSG)_2_ can also be converted to the disulfide HMA4n(SS) under the conditions; its overall concentration increased initially and then decreased once the oxidation was >80% complete (Fig. 2c–f); (3) monothiol Grxs lacking the N-terminal Cys residue are inactive but those lacking the C-terminal Cys residue are about twice as active (Fig. 3a). Consequently, the catalysis relies on the N-terminal Cys residue only, but the C-terminal Cys appears to modulate both the enzyme activity (by acting as a catalytic brake) and the catalytic pathway (see Discussion). Intriguingly, most native monothiol Grxs are inactive in the classic Grx activity assay but, in conjunction with GSH, are important in iron homeostasis and Fe–S cluster assembly.^36^

### Reduction of a protein disulfide by GSH

The GSSG/GSH ratio was adjusted to drive reaction (1) in the reverse direction (eqn (2)):2
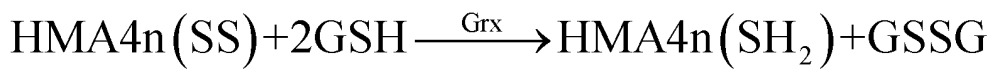



**Fig. 4:**
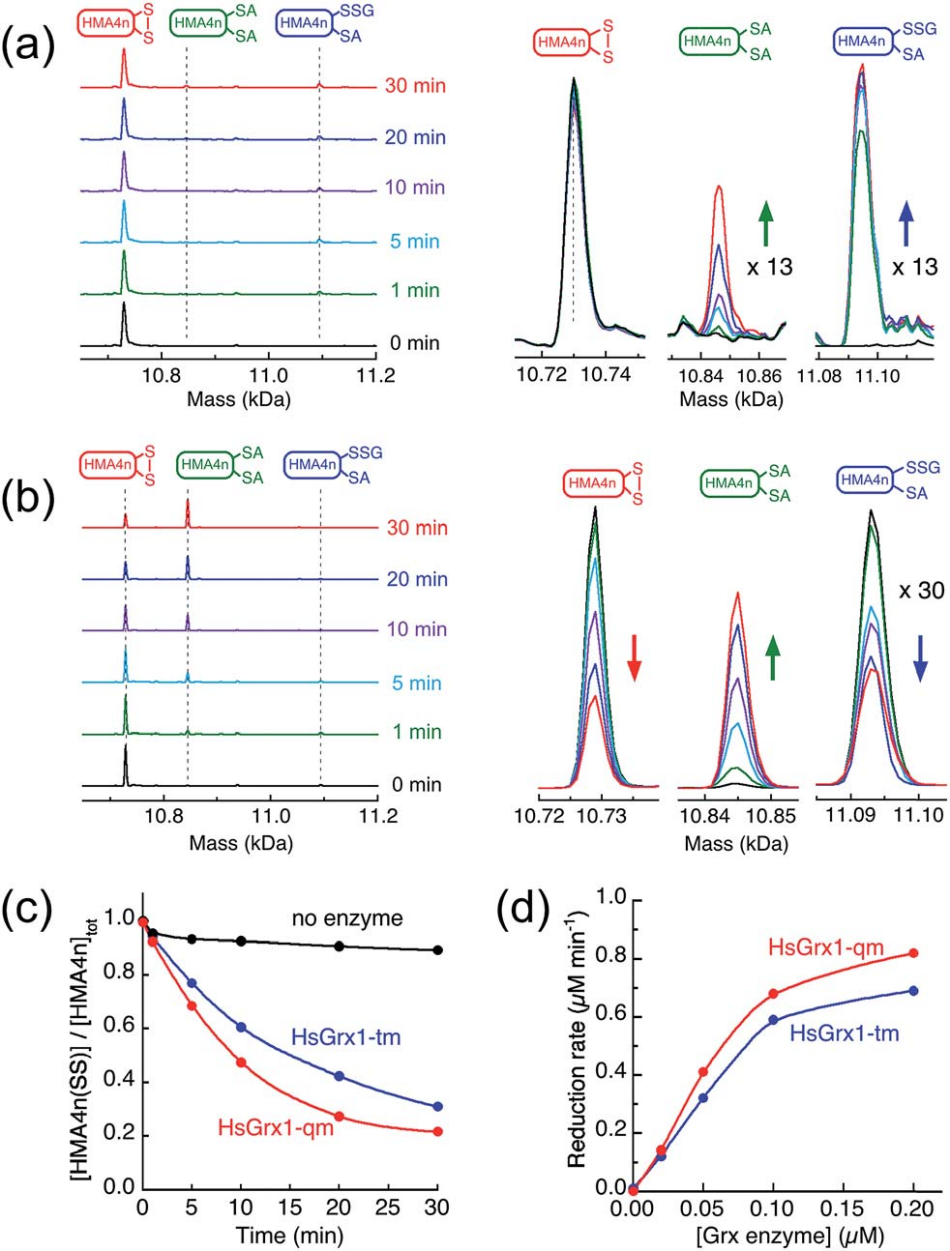
Reduction of HMA4n(SS) by GSH in KPi buffer (50 mM, pH 7.0, 100 mM NaCl): (a) IAA/ESI-MS analysis of HMA4n (10 μM) in the buffer containing GSSG (20 μM)/GSH (800 μM) without a Grx enzyme, inset: expanded view for the two minor HMA4n components; (b) the same analysis upon addition of HsGrx1-tm (100 nM) into (a); (c) reduction time course with no enzyme control and with either HsGrx1-tm or HsGrx1-qm as catalyst (each 100 nM); (d) correlation of catalytic rate with HsGrx1 enzyme concentrations (note: the catalytic rates given in Table 1 are obtained from the slopes of the best linear fits of the first three data points with enzyme concentration ≤ 0.1 μM).

Incubation of HMA4n(SS) (10 μM) in de-oxygenated KPi buffer (50 mM, pH 7.0) containing GSSG (20 μM)/GSH (800 μM) was followed by IAA/ESI-MS analysis.^35^ In the absence of enzyme, a fast exchange equilibrium was established between HMA4n(SS) and HMA4n(SH)(SSG) (∼7%; in 1–2 min). The latter was reduced further to HMA4n(SH)_2_ at a slow rate (∼0.01 μM min^–1^; Fig. 4a and c). However, upon addition of catalytic amounts of HsGrx1-tm, HMA4n(SS) was reduced enzymatically to the fully reduced form HMA4n(SH)_2_ (Fig. 4b and c). The catalytic rate increased linearly with enzyme concentration in the range 0.02–0.10 μM, allowing reliable estimation of a substrate turnover rate of ∼6 μM per min per μM per enzyme under the conditions (Fig. 4d, Table 1). This is, however, only one quarter of the substrate turnover rate for the reverse reaction (1) (Table 1) and implies that the catalytic routes and mechanisms of reactions (1) and (2) must be different.

The steady-state concentration of the intermediate species HMA4n(SH)(SSG) decreased with increased enzyme concentration, *i.e.*, decreased as the catalytic reduction rate increased (Fig. 4a and b; Table 2). This suggested that the pre-chemical formation of HMA4n(SH)(SSG) was not a bottleneck in the reduction and so other route(s) toward the final product HMA4n(SH)_2_ must exist.

**Table 2:** Catalytic reduction of P(SS) (P = HMA4n) by HsGrx1-tm^*a*^

[HsGrx1-tm] (μM)	Reduction rate of P(SS) (μM min^–1^)	Steady-state [P(SH)(SSG)] (μM)	Reductase activity (μM per min per μM per enzyme)
0	0.010(2)	∼0.7	—
0.02	0.12(1)	0.45–0.49	6.0(5)
0.05	0.32(2)	0.37–0.43	6.4(4)
0.10	0.59(2)	0.31–0.36	5.9(2)
0.20	0.69(1)	0.27–0.33	3.5(1)

^*a*^In KPi buffer (50 mM, pH 7.0) containing HMA4n(SH)_2_ (10 μM) and GSSG (20 μM)/GSH (800 μM); the bracketed values are the errors in the last digits averaged over three measurements.

Again, while the variant HsGrx1-C23S was inactive in catalysis, HsGrx1-qm and EcGrx-C14S (both retain the single N-terminal Cys residue only) were catalytically more competent, although by ∼17% only under the conditions. This contrasts with the doubling of activity for reverse reaction (1) (Table 1).

Both enzymes were detected in their fully reduced forms HsGrx1-tm(SH)_2_ and HsGrx1-qm(SH) only during catalysis under the conditions (Fig. S3†), indicating that they are the resting enzyme forms at high GSH concentration. Again, this ensures their optimal function as reductases under the conditions. We have demonstrated that, in the presence of abundant GSSG/GSH, the Grx enzymes shuttle rapidly (*t*_1/2_ < 1 min) between three oxidation states: internal disulfide Grx(SS), glutathionylated Grx(SH)(SSG) and dithiol Grx(SH)(S^–^).^16^ Grx(SH)(SSG) is the key intermediate species for the transition between Grx(SS) and Grx(SH)(S^–^) but is not stable relative to the latter two forms: it is detected maximally at 3–4% of total fractions in a solution of GSSG/GSH with a reduction potential *E*_GSSG_ (for the GSSG/2GSH couple) approaching the standard reduction potential *E*o′P(SS) for the Grx(SS)/Grx(SH)(S^–^) redox couple.^16^,^23^

### Thiol–disulfide exchange between HMA4n and Atox1

**Fig. 5:**
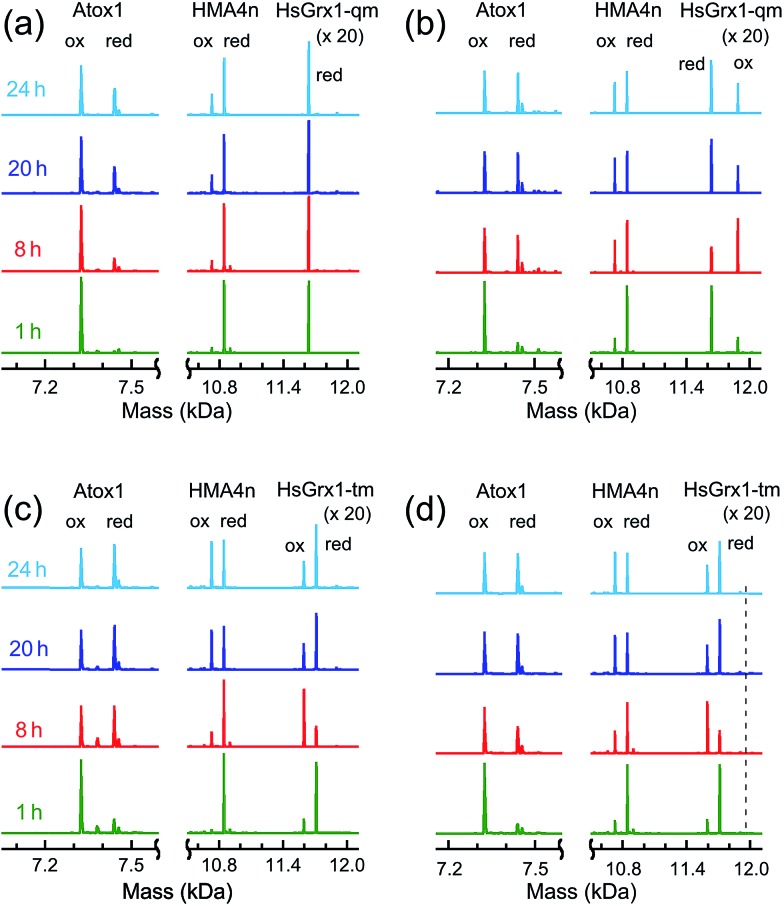
IAA/ESI-MS analysis of reaction progress and protein speciation for thiol–disulfide exchange between Atox1(SS) and HMA4n(SH)_2_ (each 10 μM) in deoxygenated Mops buffer (50 mM, pH 7.0) with either monothiol or dithiol Grx enzymes: (a) monothiol HsGrx1-qm (0.5 μM) (indistinguishable from the result with no enzyme present); (b) monothiol HsGrx1-qm (0.5 μM) plus GSH (1.0 μM); (c) dithiol HsGrx1-tm (0.5 μM); (d) dithiol HsGrx1-tm (0.5 μM) plus GSH (1.0 μM). Note: for protein dithiol, the oxidized (ox) and the reduced (red) form is P(SS) and P(SA)_2_, respectively and for protein monothiol, the oxidized (ox) and the reduced (red) form is P(SSG) and P(SA), respectively; the dashed line in (d) indicates the position for the putative species HsGrx1-tm(SA)(SSG).

HMA4n and Atox1 are both metal-binding proteins with two surface-exposed vicinal cysteinyl thiols that are the major ligands for the respective metal-binding sites (Fig. 1d and e). These thiols may be oxidized to an internal disulfide that lacks metal-binding capability.^23^,^37^ Their reduction potentials are essentially identical at ∼–190 mV (Table S1†). Thiol–disulfide exchange (eqn (3)) between these two proteins is very slow (Fig. 5a; *t*_1/2_ > 24 h) but is catalyzed by Grx enzymes (Fig. 5b and c). This provided an opportunity to investigate the behavior of Grx enzymes in the presence and absence of GSH. Each experiment was undertaken from both directions of eqn (3). The progress of the forward reaction is shown in Fig. 5 and that of the reverse reaction in Fig. S4.†

3 Atox1(SS) + HMA4n(SH)_2_ ⇌ Atox1(SH)_2_ + HMA4n(SS)

Atox1(SS) and HMA4n(SH)_2_ (each 10 μM) were mixed and incubated in de-oxygenated KPi buffer (50 mM, pH 7.0) at room temperature for 24 h. Addition of monothiol HsGrx1-qm (0.5 μM) into the system had little impact on the slow thiol–disulfide exchange rate and HsGrx1-qm was detected in its original reduced form of HsGrx1-qm(SH) only (‘red’ in Fig. 5a). Addition of catalytic GSH (1.0 μM) accelerated the exchange rate considerably (*t*_1/2_ < 8 h) and the enzyme HsGrx1-qm was detected as a mixture of HsGrx1-qm(SSG) and HsGrx1-qm(SH) (‘ox’ and ‘red’ in Fig. 5b). A control experiment in the absence of enzyme demonstrated that GSH at 1.0 μM had no detectable impact. The reduction of Atox1(SS) was coupled to the simultaneous oxidation of HMA4n(SH)_2_ (Fig. 5a and b).

In contrast, the dithiol HsGrx1-tm (0.5 μM) clearly catalyzed reaction (3) in the absence of GSH (Fig. 5c) and the enzyme was detected as a mixture of its oxidized HsGrx1-tm(SS) and reduced HsGrx1-tm(SH)_2_ forms. Inclusion of GSH (1.0 μM) into the exchange mixture had little impact on the exchange process (Fig. 5d).

These observations were mirrored by the equivalent experiments for the reverse of reaction (3) (Fig. S4†).

Reaction (3) is slow, even in the presence of a catalytic amount of a Grx enzyme. This is supported by detection of both oxidized and reduced Grxs as the resting enzyme forms consistent with existence of at least two rate-limiting steps for the catalysis (Fig. 5b–d and S4†). In each case, the Grx enzyme was added in fully reduced form. It was oxidized only partially during the catalysis to reach a maximal oxidized ratio at a reaction time of ∼8 h and then the ratio decreased slowly toward a redox equilibrium (Fig. 5b–d and S4b†). This means that Grx cannot function as an effective catalyst without the driving force provided by the abundant cellular components GSH/GSSG as electron donor/acceptor. Apparently, reaction (3) is too slow to be physiologically relevant but can be analyzed (see Discussion) with conclusions that illuminate the various reaction mechanisms in molecular detail.

## Discussion

### General considerations for thiol–disulfide exchange reactions

These are second-order reactions that involve sequential bimolecular nucleophilic substitution (S_N_2), expressed generally in eqn (4):^6^,^14^

4 RS_nuc_^–^ + RS_c_ – S_lg_R → RS_nuc_ – S_c_R + RS_lg_^–^

RS_nuc_^–^ is the attacking thiolate, RS_c_ is the central sulfur participating in both reactant and product disulfide and RS_lg_^–^ is the leaving group. The attacking thiolate may be pre-existent or generated dynamically *in situ* by deprotonation, depending on its p*K*_a_ and the environments of the thiol and the disulfide. The attack can only occur along the direction of the disulfide bond.^6^,^14^ Reaction is generally slow for aliphatic thiols and disulfides. GSH is a weak nucleophile (due to the high thermodynamic barrier involved in deprotonating its cysteinyl thiol: p*K*_a_ ∼ 8.5), while GSSG is a weak electrophile and GS a poor leaving group.^11^ Consequently, reactions (1)–(3) normally require a redox enzyme.^38^ The thioredoxin family have evolved to act as both excellent nucleophiles and leaving groups. In addition, their solvent-exposed reactive thiolates can act as accessible electrophilic central sulfur atoms to be targeted in disulfide forms and so these enzymes promote thiol–disulfide exchange reaction (4). A bonus for Grx enzymes is their capacity to employ the GSSG/2GSH redox couple as both catalytic cofactor and co-substrate. These general considerations rationalize the catalytic mechanisms discussed below.

### Distinct monothiol and dithiol mechanisms for thiol–disulfide exchange

**Scheme 2:**
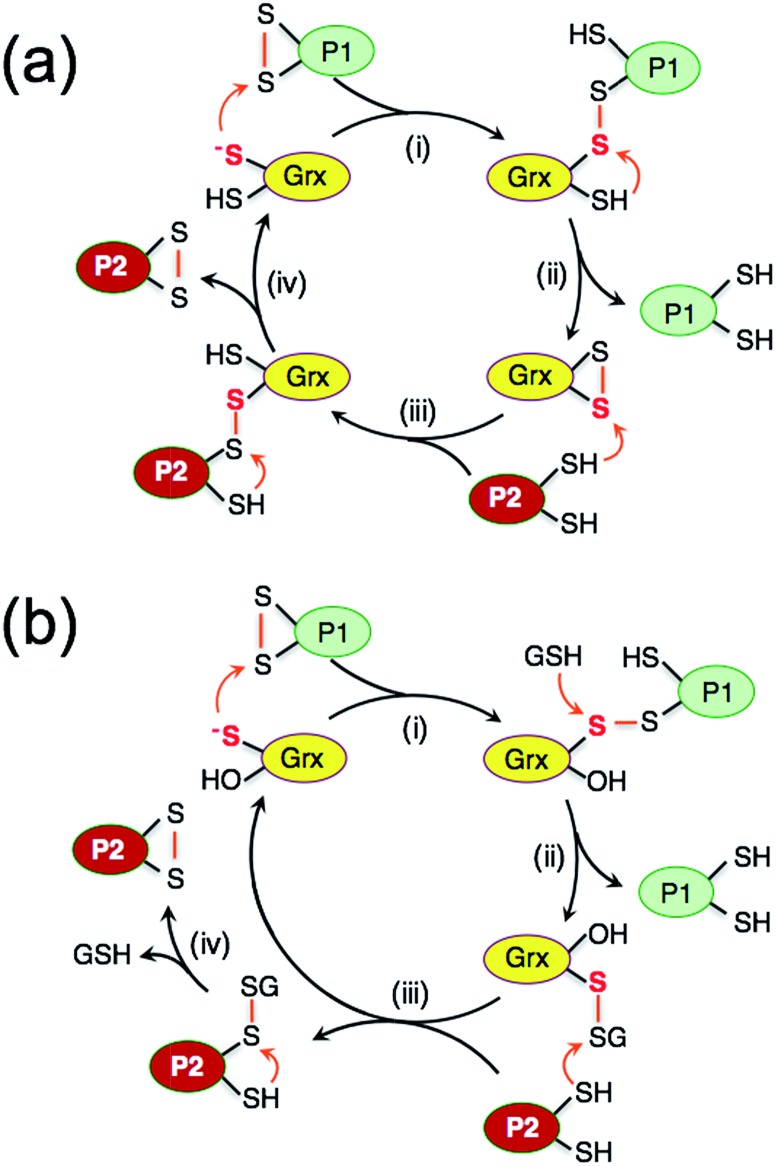
Dithiol mechanism in the absence of GSH (a) and monothiol mechanism in the presence of GSH (b) employed by Grx enzymes catalyzing thiol–disulfide exchange between proteins P1(SS) and P2(SH)_2_.

The uncatalyzed rate of the thiol/disulfide exchange between HMA4n and Atox1 (eqn (3)) is very slow. In the absence of GSH, the exchange in either direction is catalyzed by the dithiol HsGrx1-tm (Fig. 5c and S4b†), but not by the monothiol HsGrx1-qm (Fig. 5a and S4a†). This is consistent with the dithiol mechanism of Scheme 2a: catalysis proceeds sequentially *via* two thiol–disulfide exchange reactions with the requirement for both cysteinyl thiols in the active site. As required by this model, HsGrx1-tm was detected to shuttle between the two active forms HsGrx1(SH)(S^–^) and HsGrx1(SS) (Fig. 5c and S4b†) whereas the inactive monothiol HsGrx1-qm remained as HsGrx1(OH)(S^–^) only (Fig. 5a and S4a†). Notably, during the catalysis, the relative concentration of HsGrx1(SS) increased with time to reach a maximum at ∼8 h and then decreased again towards a redox equilibrium and such behavior was seen for either direction of eqn (3) (Fig. 5c and S4b†). This indicates that step (i) of Scheme 2a is faster than step (iii) due to the more favorable nucleophilic attack on a disulfide bond by a Grx thiolate anion relative to a neutral protein thiol.

On the other hand, the presence of a catalytic amount of GSH induced activity in the monothiol HsGrx1-qm, with comparable efficiency (Fig. 5b). This is consistent with the monothiol mechanisms of Scheme 2b. Step (i) produces Grx(OH)(SS)(SH)P1 which cannot by itself release the protein P1 as the fully reduced form P(SH)_2_. Addition of GSH facilitates key step (ii) to allow the monothiol Grx to be glutathionylated as Grx(OH)(SSG) with simultaneous release of the fully reduced P1(SH)_2_. This scheme is supported by the direct detection of the two catalytically competent enzyme forms Grx(OH)(S^–^) and Grx(OH)(SSG) of the monothiol Grx throughout the catalytic process (Fig. 5b). Apparently, step (ii) is promoted by the specific interaction between GSH and the Grx enzyme (Fig. 1b). In support, protein P2(SH)_2_ (HMA4n(SH)_2_ in Fig. 5b) is unable to promote step (ii) due to lack of a specific binding interaction and to the steric hindrance of forming a transient complex between three protein molecules.

The oxidized enzyme form Grx(OH)(SSG) may induce P2(SH)_2_ oxidation *via* steps (iii) and (iv). Step (iii) was demonstrated by our recent observation that Grx(OH)(SSG) reacts with a protein monothiol P(SH) to yield P(SSG) only^16^ while step (iv) is a spontaneous non-catalytic chemical process.

Similar to the reactions in Fig. 5c (Scheme 2a), the oxidized enzyme form Grx(OH)(SSG) in Fig. 5b (Scheme 2b) was also detected with the highest relative concentration at a reaction time of ∼8 h, suggesting that step (i) is faster than step (iii) in both cases for the reason mentioned above.

On the other hand, both steps (i) and (iii) are rate-limiting (relative to steps (ii) and (iv)) in both the monothiol and dithiol mechanisms of Scheme 2a and b. This rationalizes the detection of two forms of each enzyme throughout the catalytic exchange reactions and the slow catalytic processes (Fig. 5b–d and S4b†).

It was speculated that oxidation of P(SH)_2_ by Grx(SH)(SSG) might also proceed *via* nucleophilic attack of P(SH)_2_ on the reactive sulfur atom in Grx(SH)(SSG) (similar to step (iii) of 2a)^39^ and this will lead to an alternative reaction Scheme S1.† However, such speculation is not supported by the experimental data (see further discussion in the ESI†).

### The mechanisms of catalytic oxidation of protein dithiols P(SH)_2_ by GSSG

**Scheme 3:**
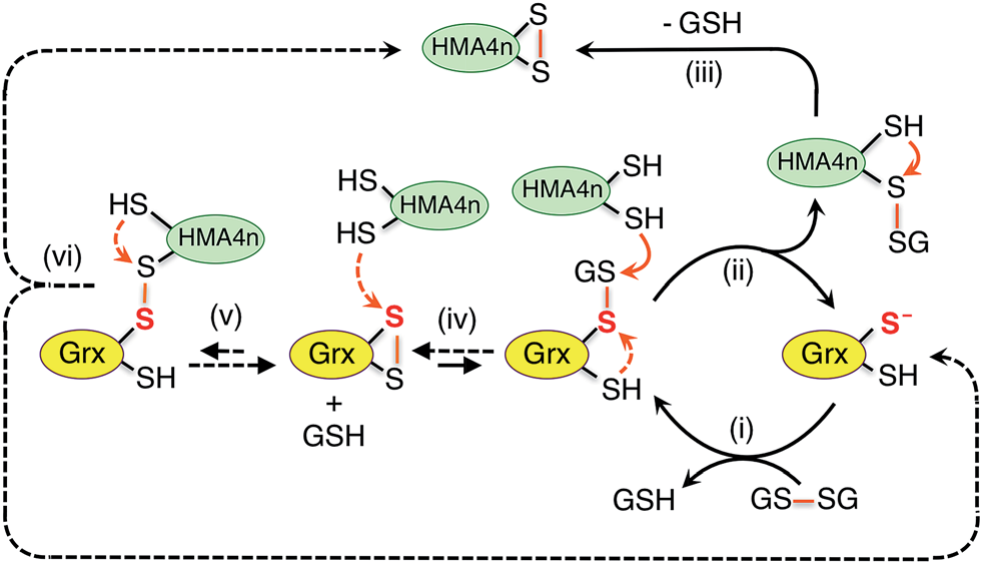
Proposed parallel monothiol–dithiol mechanism for catalytic oxidation of protein dithiol P(SH)_2_ (such as HMA4n(SH)_2_) by Grx/GSSG. The monothiol oxidation route is shown in solid arrows whereas the dithiol oxidation route in dashed arrows. Under the oxidative conditions, the resting enzyme forms for dithiol Grx and monothiol Grx are Grx(SS) and Grx(SSG), respectively.

The above discussion provides a solid basis for interpretation of the experimental data of Fig. 2 in terms of Scheme 3. Substrate HMA4n(SH)_2_ (*E*o′P(SS) = –192 mV; Table S1†) is thermodynamically unstable in a buffer at pH = 7.0 containing GSSG (400 μM)/GSH (40 μM): ∼–81 mV (eqn (S1) and (S2); see ESI†). In the absence of a catalyst, the oxidation can only be initiated by nucleophilic attack of a thiol in HMA4n(SH)_2_ on GSSG. This, however, is a kinetically unfavorable process due to the poor nucleophilicity of a normal protein thiol group and the relative stability of the disulfide bond in GSSG.

Addition of enzyme allows effective activation of the GSSG disulfide bond to yield active Grx(SH)(SSG) (step i). Parallel routes then become available. The first is a monothiol mechanism (shown in solid arrows) in which the enzyme directly glutathionylates a protein thiol in HMA4n(SH)_2_ to produce unstable HMA4n(SH)(SSG) (step ii) that converts spontaneously to HMA4n(SS) (step iii).^40^ The second is a dithiol mechanism (in dashed arrows) in which Grx(SH)(SSG) oxidizes spontaneously to the disulfide Grx(SS) (step iv). The latter may execute the oxidation of protein dithiol substrate *via* steps (v) and (vi).

Evidence for the dithiol mechanism includes: (1) under the oxidative conditions imposed by GSSG, the Grx enzyme was detected as the fully oxidized form Grx(SS) only (Fig. 3b): it is the resting enzyme form under these conditions and is consistent with both reverse step (iv) and forward step (v) being rate-determining steps in Scheme 3; (2) in the absence of GSSG/GSH, dithiol HsGrx1-tm, but not monothiol HsGrx1-qm, catalyzed the thiol–disulfide exchanges of eqn (3) (Fig. 5 and S4†). These can proceed *via* a dithiol mechanism only, as outlined in Scheme 2a.

Evidence for the monothiol mechanism in the presence of GSSG/GSH is summarized below:

(1) Intermediate species HMA4n(SH)(SSG) was readily detected, especially in the early stages when its production rate reached a maximum due to the high relative concentration of substrate HMA4n(SH)_2_ (Fig. 2c–f). This intermediate can only be generated *via* step (ii) of the monothiol route.^41^

(2) Mutation of the C-terminal Cys to Ser in the Cys–xx–Cys active site motif of dithiol Grx enzymes eliminated the possibility of a dithiol mechanism and enhanced the catalytic activity by ∼100% (Table 1). This confirms the existence of a monothiol route that is more efficient than the dithiol route. In support, Grx(OH)(SSG) was detected as the resting enzyme form (Fig. 3b).^42^

(3) In the absence of GSH, monothiol HsGrx1-qm was not able to catalyze reaction (3), the thiol–disulfide exchange between two dithiol proteins. However, addition of a catalytic quantity of GSH rescued its catalytic activity and the enzyme was detected to shuttle between the two catalytically competent forms HsGrx1-qm(SH) and HsGrx1-qm(SSG) during the catalysis (Fig. 5a and b). This can only occur *via* the monothiol steps of Scheme 2b.

These observations demonstrate that the trace enzyme species Grx(SH)(SSG), although difficult to detect under oxidizing conditions, plays an important role in oxidation of protein dithiols P(SH)_2_*via* the monothiol mechanism. This may be attributed to the high efficiency of its glutathionylation of protein thiols (step ii of Scheme 3): the fully reduced Grx enzyme is an excellent leaving group upon reduction of a disulfide bond involving its reactive thiol (see eqn (4)).

As discussed above, speculation that the oxidation of HMA4n(SH)_2_ might proceed also *via* nucleophilic attack of HMA4n(SH)_2_ on the reactive sulfur atom in Grx(SH)(SSG)^39^ is not supported by our previous equivalent experiments with protein monothiol P(SH) (see further discussion in ESI†).^16^ Nevertheless, reaction Scheme 3 demonstrates that Grxs are a class of versatile enzymes with flexibility in catalyzing protein dithiol oxidation by GSSG *via* either a monothiol mechanism or a dithiol mechanism or, more likely, both in parallel.

### The mechanism of catalytic reduction of protein disulfides P(SS) by GSH

An enzyme normally catalyzes a reaction in both directions. However, catalytic oxidation of a protein dithiol by Grx/GSSG (eqn (1)) may proceed *via* two different routes as shown in Scheme 3. Step (iii) in the key monothiol route does not require the action of an enzyme. This raises an intriguing question: what is the reaction mechanism for the reverse catalytic process, *i.e.*, for catalytic reduction of a protein disulfide by Grx/GSH, as represented by eqn (2). This has been controversial and three mechanisms (Scheme 1a–c) have been proposed and are debated currently. The availability of IAA/ESI-MS analysis in this work allowed design of a number of experiments to examine the possibilities.

#### (a) Chemical pre-equilibrium monothiol mechanism

This mechanism is adapted from that proposed for the classic HEDS assay (Scheme 1a).^21^,^22^ It assumes that there is a fast pre-equilibrium between protein disulfide P(SS) and GSH to generate disulfide P(SH)(SSG) that is reduced enzymatically by Grx *via* the monothiol mechanism. This scheme was used recently to describe the mechanism of catalytic reduction of a disulfide bond in both human SOD1 and human Trx1 by Grx/GSH.^30^,^31^ In fact, it is equivalent to the proposal that step (iii) in Scheme 3 is chemically reversible and rapidly equilibrated. If this was the case, the reduction of HMA4n(SS) would proceed, at elevated GSH concentration, *via* reverse steps (iii) → (ii) → (i).

Experimental evidence from the present work appears to support this mechanism: in a buffer containing GSSG (20 μM)/GSH (800 μM) without a Grx enzyme, HMA4n(SS) (10 μM) does equilibrate quickly with GSH to yield HMA4n(SH)(SSG) (∼7%) that, in the absence of a catalyst, is reduced at a very slow rate to HMA4n(SH)_2_ (Fig. 4a and c). Upon addition of an active Grx enzyme, the reduction rate increased considerably and was proportional to the enzyme concentration (Fig. 4b and c). However, the steady-state concentration of HMA4n(SH)(SSG) decreased proportionally with increase in the reduction rate (Fig. 4a and b; Table 2). This indicates that this chemical pre-equilibrium adjusts too slowly to account for the observed enzyme activity and that there must be parallel reduction routes in operation. Similar conclusions were reached from a recent re-evaluation of the reaction mechanism of the HEDS assay (Scheme 1a).^33^

#### (b) Parallel monothiol–dithiol mechanisms

**Scheme 4:**
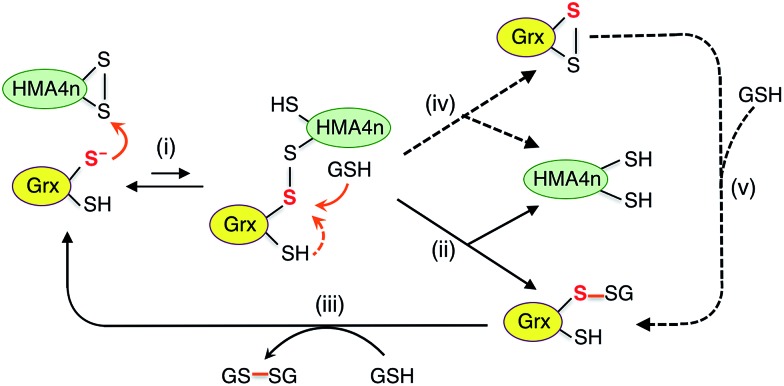
Proposed parallel monothiol–dithiol mechanisms for catalytic reduction of protein disulfides P(SS) by Grx/GSH. The monothiol reduction route is shown in solid arrows with the dithiol route in dashed arrows. Under the reducing conditions, the resting forms for dithiol and monothiol Grx enzymes are Grx(SH)(S^–^) and Grx(OH)(S^–^), respectively.

The catalytic reduction of a protein disulfide by Grx/GSH may proceed *via* the reverse direction of step (vi) of Scheme 3. This would lead to the parallel monothiol–dithiol mechanisms shown in Scheme 4.^23^ This possibility is now explored.

Scheme 4 is actually an integration of the two separate mechanisms (mono- and di-thiol) presented in Scheme 1b and c. It suggests that dithiol Grx enzymes are adapted to catalyze protein disulfide reduction by GSH *via* direct attack on the disulfide to form an enzyme–protein disulfide complex (step i). This is followed by either a monothiol mechanism (steps ii, iii) or a dithiol mechanism (step iv) or both in parallel.

Step (i) is seen to occur in Fig. 5 (see Scheme 2) as the first step of each catalytic reaction and is supported by our direct detection of the transient enzyme–protein complex in a similar case.^23^ Step (ii) is the key for the monothiol mechanism in which the protein mixed disulfide bond is attacked by an external GSH molecule to complete the reduction and to yield the glutathionylated enzyme Grx(SH)(SSG). This step is promoted by the specific interaction between GSH and Grx (Fig. 1b) and by the high concentration of GSH. On the other hand, step (iv) of the dithiol mechanism is independent of the GSH concentration and involves formation of the enzyme disulfide Grx(SS). Both Grx(SH)(SSG) and Grx(SS) can be re-activated by GSH at high concentration.^16^

Mutation of the C-terminal Cys to Ser in both HsGrx1 and EcGrx1 led to ∼17% increase in their activities for reduction of HMA4n(SS) by GSH (eqn (2)). This contrasts with ∼100% increase in their oxidase activities for oxidation of HMA4n(SH)_2_ by GSSG (eqn (1); Table 1). The reasons could be twofold: (1) GSH at the high concentrations required for disulfide reduction is expected to promote the monothiol reduction route for dithiol Grx enzymes; (2) the Grx(SS) species formed *via* the dithiol mechanism can be re-activated efficiently by GSH at high concentrations.^16^ Nevertheless, the C-terminal Cys acts as a catalytic brake in either case.

Notably, the catalytic rate for reduction of HMA4n(SS) is slower than that of the reverse reaction by the same enzyme (about 24% and 15% for dithiol and monothiol Grx, respectively; see Table 1). The reason is that the oxidation is promoted by a fast non-enzymatic step (iii) in Scheme 3 whereas the reverse reaction rate of this step is much slower, even at the high GSH concentration of 800 μM (Fig. 4a).

The resting enzyme form now detected is fully reduced Grx(SH)(S^–^) or Grx(OH)(S^–^) (Fig. S3†), consistent with the rate-determining step (i) at high GSH concentration in Scheme 4.

It has been reported that both active site Cys residues in EcGrx1 are required for catalytic reduction of certain disulfides (see detailed discussion in ESI†).^21^,^32^,^43^–^45^ The conclusion was that the C-terminal Cys in dithiol Grxs does not just simply act as a catalytic brake, it also plays an important role in catalysis when the monothiol route is blocked. Indeed, the presence of two Cys residues in the Grx active site allows the versatility of access to both the monothiol and dithiol pathways. However, for catalytic oxidation/reduction of a surface-exposed protein dithiol/disulfide such as those in HMA4n and Atox1 (Fig. 1d and e), the monothiol mechanism is more efficient for both HsGrx1 and EcGrx1 (see Fig. 3a and 4c and d).

## Conclusions

Grxs are a class of GSH-dependent thiol–disulfide oxidoreductases that directly bridge the reversible sulfur redox chemistry of protein thiols to the abundant cellular non-protein GSSG/2GSH redox couple. They are adapted to catalyze oxidation of a protein dithiol to a disulfide by GSSG *via* either a monothiol or a dithiol mechanism or more likely, both in parallel (Scheme 3). The monothiol mechanism is more efficient than the dithiol mechanism, likely due to the fact that the glutathionylated substrate species P(SH)(SSG) generated *via* the monothiol mechanism (step ii) is highly activated and converted rapidly and spontaneously to the stable disulfide product (steps iii).

Grxs are also adapted to catalyze reduction of protein disulfides by GSH *via* parallel monothiol–dithiol mechanisms (Scheme 4). The monothiol mechanism relies on access of GSH to the mixed disulfide of the Grx–protein complex. When access is inhibited, the reduction is constrained to the dithiol mechanism. A chemical pre-equilibrium monothiol mechanism may also play a minor role but this non-catalytic process is generally too slow for fast enzyme action. In addition, Grxs can catalyze reversible glutathionylation/deglutathionylation *via* a monothiol mechanism.^16^ The flexibility of Grx enzymes in catalysis can be attributed primarily to the specific binding interaction between GSH and the enzymes (Fig. 1b).

Recent progress shows that the relative concentrations of GSH and GSSG vary considerably across different cellular compartments and, consequently, so do the formal reduction potentials imposed by the GSSG/2GSH redox couple.^29^ However, cellular processes usually work under steady-state kinetic controls that deviate from thermodynamic equilibria.^46^,^47^ In this sense, Grx enzymes must play important roles in facilitating essential dynamic cellular redox processes.

## Experimental section

### Materials and general methods

General chemicals and reagents were purchased from Sigma-Aldrich and used as received. Stock solutions of dithiothreitol (DTT) and GSH were prepared in deoxygenated Milli-Q water and stored in an anaerobic glove box. Their concentrations based on quantitative dissolution were confirmed and calibrated with the Ellman assay.^48^

To ensure accurate quantification of redox events and redox equilibria, all chemicals and proteins in air-sensitive reduced forms were prepared, stored and handled under anaerobic conditions inside a glove-box. Unless indicated, most experiments on thiol–disulfide exchange reactions were also conducted under anaerobic conditions using thoroughly-deoxygenated buffers.

### Protein production, quantification and characterization

Various Grx enzymes and Atox1 protein were expressed and isolated as reported.^16^,^23^ The protein domain HMA4n was also expressed and isolated according to a previous report.^34^ Each purified protein was fully reduced with DTT and buffer-exchanged using a desalting column with thoroughly deoxygenated Mops buffer (50 mM, pH 7.0, 100 mM NaCl) in an anaerobic glove-box. Correct thiol content was determined in each case with Ellman's reagent 5,5-dithiobis(2-nitrobenzoic acid) (DTNB) based on the concentrations estimated from respective solution absorbance at 280 nm.^48^ The oxidized proteins HMA4n(SS), Atox1(SS), HsGrx1-tm(SS) and EcGrx1(SS) were generated by oxidation with a slightly excess of [Fe^III^(CN)_6_]^3–^ and then buffer-exchanged with a desalting column to separate the protein component from the Fe complexes. The reduction potentials of protein disulfides were determined as reported (see ESI†).^16^

### Electrospray ionization mass spectrometry

All experiments were conducted on an Agilent time-of-flight mass spectrometer (TOF-MS) (model 6220, Palo Alto, CA) coupled to an Agilent 1200 LC system with details given previously.^23^ Control experiments demonstrated that, for the same protein, the integrated mass spectral intensities for different redox forms are proportional to their relative concentrations, as demonstrated previously.^23^ Consequently, the fraction of each oxidation component for the same protein was determined by integration of the mass spectral peak area of that component and then divided by the sum of the total peak areas for all components of that protein. All protein thiols were alkylated with excess iodoacetamide (IAA) for ESI-MS detection. For each thiol group, a net mass of 57 Da for an acetamide group –CH_2_CONH_2_ was added to the detected molar mass of the protein target.

### Oxidation of protein dithiols in GSSG/GSH buffer

The oxidation was effected by incubation of HMA4n(SH)_2_ (10 μM) in a redox buffer composed of GSSG (400 μM)/GSH (40 μM) in KPi buffer (50 mM, pH 7.0) in the absence and presence of a selected Grx enzyme at various concentrations (20–100 nM). The reactions were followed by IAA/ESI-MS analysis and were started by adding HMA4n(SH)_2_ containing either no Grx enzyme or various amounts of a selected Grx enzyme into the GSSG/GSH redox buffer to make a reaction mixture containing the required final concentration for each component as quoted above. An aliquot of the reaction mixture (∼10 μL) was transferred into a micro-tube containing an IAA solution (5 μL 50 mM in H_2_O; >50-fold excess) to quench the reaction along the reaction time course. The oxidation rate for each reaction was calculated from the initial reaction rate that increased linearly with time. The substrate turnover numbers for each enzyme expressed as μM per min per μM per enzyme was quoted from the reaction with an enzyme concentration at 20 nM. The background oxidation rate was removed in the calculation.

### Reduction of protein disulfides in GSSG/GSH buffer

HMA4n(SS) with an internal disulfide was the sole product of HMA4n(SH)_2_ oxidation by [Fe^III^(CN)_6_]^3–^ and was selected for the experiments. The reduction was effected by incubation of HMA4n(SS) (10 μM) in a redox buffer composed of GSSG (20 μM)/GSH (800 μM) in KPi buffer (50 mM, pH 7.0) in the absence or presence of a selected Grx enzyme at various concentrations (20–200 nM). The reactions were followed by IAA/ESI-MS analysis. The control reaction with no enzyme was started by adding HMA4n(SS) into the GSSG/GSH redox buffer. The catalytic reaction was started by first addition of HMA4n(SS), followed by the required Grx enzyme. The reaction was timed upon the final addition of the enzyme and was followed by IAA/ESI-MS analysis as detailed above.

### Thiol–disulfide exchange between HMA4n and Atox1

These two proteins, one in oxidized form P1(SS) and the other in reduced form P2(SH)_2_, were mixed in 1 : 1 molar ratio (each 10 μM) in de-oxygenated KPi buffer (50 mM, pH 7.0), followed by adding no enzyme or selected Grx enzyme (0.05 μM) to start the exchange reaction. GSH (1.0 μM) was included for selected reactions. The reaction was conducted from either direction and followed by IAA/ESI-MS analysis.

## Conflicts of interest

There are no conflicts to declare.

## Supplementary Material

Supplementary information
