# Supplementary material for: Glutaredoxins employ parallel monothiol–dithiol mechanisms to catalyze thiol–disulfide exchanges with protein disulfides
Source: Chem Sci. 2017 Dec 6;9(5):1173–83. doi: 10.1039/c7sc04416j (PMC5885593; doi:10.1039/c7sc04416j)
Supplement: Supplementary file 1 [file SC-009-C7SC04416J-s001.pdf]

Electronic Supplementary Material (ESI) for Chemical Science.
This journal is © The Royal Society of Chemistry 2017

Glutaredoxins Employ Parallel Monothiol-Dithiol Mechanisms to Catalyze Thiol-Disulfide Exchanges for Protein Disulfides

Ashwinie A. Ukuwela,[†] Ashley I. Bush,[‡] Anthony G. Wedd[†] and Zhiguang Xiao[†,‡,*]

[†] School of Chemistry and Bio21 Molecular Science and Biotechnology Institute, The University of Melbourne, Parkville, Victoria 3010, Australia.
[‡] Melbourne Dementia Research Centre, Florey Institute of Neuroscience and Mental Health, The University of Melbourne, Parkville, Victoria 3052, Australia.

Email: zhiguang.xiao@florey.edu.au

Tel: +61 3 9035 6072

Supporting Information

Determination of reduction potentials for protein disulfides

The reduction potentials of Atox1(SS) and Grx(SS) have been determined,[1] but that of HMA4n(SS) has not. This work determined the reduction potential of the latter following the same two protocols.[1] The first was the poised potential method that employed GSSG/2GSH as a redox buffer. HMA4n (~10 μM) was incubated overnight under anaerobic conditions in a series of redox buffers GSSG/GSH (total [GSH + 2 GSSG] = 10 mM) in KPi (50 mM, pH 7.0) whose reduction potentials are defined by eqs S1-4. The second was redox equilibration between two protein dithiols with different starting oxidation states based on eqs S5-6. The protein compositions at each redox equilibrium was quantified by IAA/ESI-MS analysis.

$$GSSG + 2\,H^{+} + 2\,e^{-} \rightleftharpoons 2\,GSH \qquad \text{S1}$$

$$E'_{GSSG} = E^{o'}_{GSSG} - \frac{RT}{2F}\ln\frac{[GSH]^2}{[GSSG]} \qquad \text{S2}$$

$$P(SS) + 2\,H^{+} + 2\,e^{-} \rightleftharpoons P(SH)_2 \qquad \text{S3}$$

$$E'_{P(SS)} = E^{o'}_{P(SS)} - \frac{RT}{2F}\ln\frac{[P(SH)_2]}{[P(SS)]} \qquad \text{S4}$$

$$P1(SS) + P2(SH)_2 \rightleftharpoons P1(SH)_2 + P2(SS) \qquad \text{S5}$$

$$E^{o'}_{P1(SS)} - E^{o'}_{P2(SS)} = \frac{RT}{2F}\ln\frac{[P1(SH)_2]\,[P2(SS)]}{[P1(SS)]\,[P2(SH)_2]} \qquad \text{S6}$$

The results are summarised in Figure S1 and Tables S1-2. A standard reduction potential of $E^{o'}_{P(SS)}$ = −192 mV at pH 7.0 was determined (Figure S1). It is essentially identical to that of Atox1 ($E^{o'}_{P(SS)}$ = −191 mV), but is about 23 mV more negative than those of the two dithiol Grx enzymes (Table S1). These differences were confirmed by a series of pairwise redox equilibrium experiments (Table S2; see also Figure 5).

Further discussion of reaction Scheme IIb

An alternative route for oxidation of $P2(SH)_2$ in Scheme IIb may proceed via steps (iii) and (iv) shown in Scheme S1 below, namely, dithiol protein $P2(SH)_2$ may undertake a nucleophilic attack on the mixed disulfide bond in Grx(OH)(SSG) from the more positive sulfur group in Grx (step (iii); similar to step (iii) in Scheme IIa). This will lead to formation of an enzyme-protein complex Grx(OH)(SS)(SH)P2 which is expected to undertake further disulfide bond re-shuttling to release fully oxidized P2(SS) and fully reduced Grx(OH)(S^-) via step (iv). This possibility was speculated in a previous study.[2] However, such speculation is not supported by our recent experiments that demonstrated that reaction between monthiol Grx(OH)(SSG) and monothiol protein P(SH) yields P(SSG) only with no detectable Grx(OH)(SS)P.[1] It is likely that the sulfur atom of the GS group in Grx(OH)(SSG) is a more favourable nucleophilic target in this case due to steric reason.

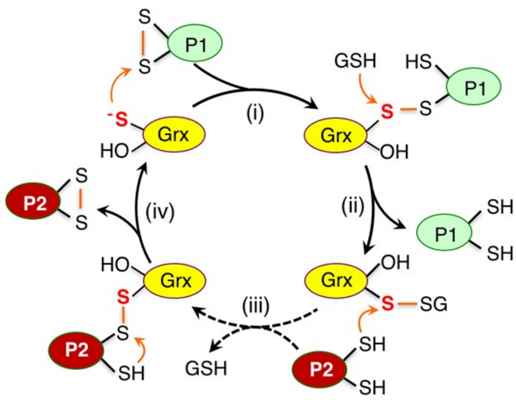

Scheme S1. Putative alternative reaction routes for $P2(SH)_2$ oxidation for Scheme IIb.

Discussion on literature examples of dithiol mechanism for EcGrx1

It has been reported that both active site Cys residues in EcGrx1 are required for catalytic reduction of certain disulfides. For example, mutation of the C-terminal Cys[14] to Ser in EcGrx1 led to a complete loss of its activity for reduction of a protein disulfide bond in ribonucleotide reductase or in bovine pancreas insulin.[3,4] This is likely due to limited access of the extenal GSH to the EcGrx1-protein disulfide bond. Indeed, an NMR structural study of catalytic GSH reduction of ribonucleotide reductase revealed that a mixed disulfide bond was formed between the EcGrx1-C14S variant and a peptide of the interacting subunit B1. The B1 peptide occupied the GSH binding site in EcGrx1 and protected the mixed disulfide bond from attack by an external nucleophile. Consequently, the monothiol mechanism facilitated by GSH was supressed.[5] A similar case was reported for GSH reduction of a protein disulfide bond in the *E. coli* enzyme 30-phosphoadenosine 50-phosphosulfate (PAPS) reductase that was catalyzed by EcGrx1, but not by EcGrx1-C14S.[6] A systematic study and analysis suggested that favorable electrostatic and complementary surface interactions between the protein partners are the key determinants that impose a dithiol mechanism in these cases.[7] The conclusion was that the C-terminal Cys in dithiol Grxs does not just simply act as a catalytic brake, it also plays an important role in catalysis when the monothiol route is blocked. However, for catalytic oxidation/reduction of a surface-exposed protein dithiol/disulfide such as those in HMA4n and Atox1 (Fig. 1d,e), the monothiol mechanism is more efficient for both HsGrx1 and EcGrx1 (see Figs. 3a, 4c,d).

Table S1. Reduction potentials for monothiol and dithiol sites

Protein	Organisms	Active site	pK_a [a]	$E^{o'}$ (mV) [b]	ref
dithiol					
EcGrx1	*E. coli*	**Cys^{11}**-Pro-Tyr-Cys^{14}	< 5.0 [8]	−168	1
HsGrx1-tm	*H. sapies*	**Cys^{23}**-Pro-Tyr-Cys^{26}	3.5 [9]	−169	1
Atox1	*H. sapiens*	Cys^{12}-Gly-Gly-**Cys^{15}**	5.5 [10]	−191	1
HMA4n	*A. thaliana*	Cys^{27}-Cys^{28}-x-x-Glu^{31}	−	−192	This work
monothiol					
EcGrx1-C14S	*E. coli*	**Cys^{11}**-Pro-Tyr-Ser^{14}	−	−213	1
HsGrx1-qm	*H. sapiens*	**Cys^{23}**-Pro-Tyr-Ser^{26}	−	−230	1

[a] Refer to Cys thiol highlighted in bold in the active site; [b] Determined in GSSG/GSH buffer (10 mM) based on $E^{o'}_{GSSG}$ = −240 mV (*vs* SHE) at pH 7.0 (see Figure S1).

Table S2. Comparison of $\Delta E_{P1-P2}^{o'}$ for protein dithiols determined via direct pair-wise protein-protein interaction and via separate reactions with GSSG/GSH redox buffer [a]

Equilibrium of eq 6a		K_{ex}	$\Delta E_{P1-P2}^{o'}$ (mV) from	
P1(SS)	P2(SH)$_2$		eq S6 directly	eq S1-4 indirectly
EcGrx1(SS)	HMA4n(SH)$_2$	5.07	21	24
HsGrx1-tm(SS)	HMA4n(SH)$_2$	5.31	21	23
Atox1(SS)	HMA4n(SH)$_2$	1.34 [b]	4	1
	HMA4n(SH)$_2$	0.16 [c]	−24	1
HMA4n(SS)	Atox1(SH)$_2$	0.98 [b]	−4	−1
	Atox1(SH)$_2$	0.22 [c]	−18	−1

[a] Each pair of proteins (each 10 μM) was incubated in deoxygenated KPi buffer (50 mM, pH 7.0) for 24 h and then the protein compositions were analyzed by IAA/ESI-MS approach (see Figure 2); [b] A catalytic amount of a dithiol Grx enzyme such as HsGrx1-tm or EcGrx1 (each 0.5 μM) was added to catalyze the thiol-disulfide exchange reaction between Atox1 and HMA4n; [c] Non-equilibrium position after 24 h incubation with no added Grx enzymes or in the presence of a monothiol Grx enzyme such as HsGrx1-qm.

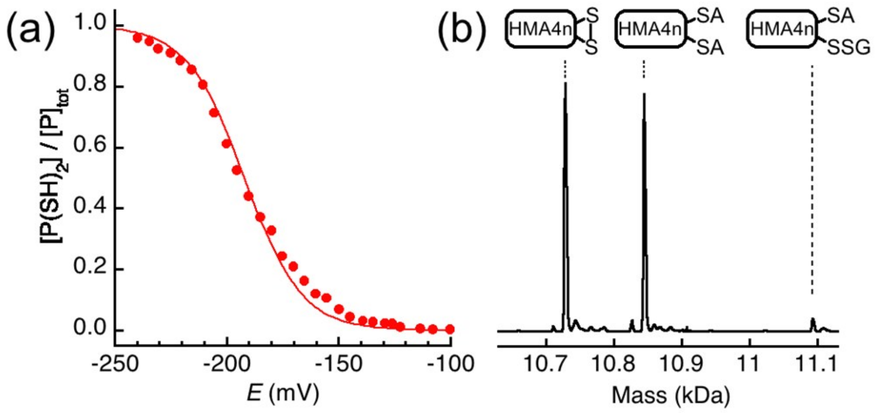

Figure S1. Determination of reduction potential for P(SS)/P(SH)$_2$ (P = HMA4n): (a) Variation of P(SH)$_2$/[P]$_{tot}$ with solution reduction potentials defined by GSSG/GSH according to eqs S1-2. The solid trace is the curve-fitting of the experimental data to eq S4 for the redox equilibrium of eq S3; (b) A representative IAA/ESI-MS spectrum detecting the redox equilibrium S3 in a redox buffer GSSG (1.85 mM)/GSH (6.3 mM) (E, −191 mV).

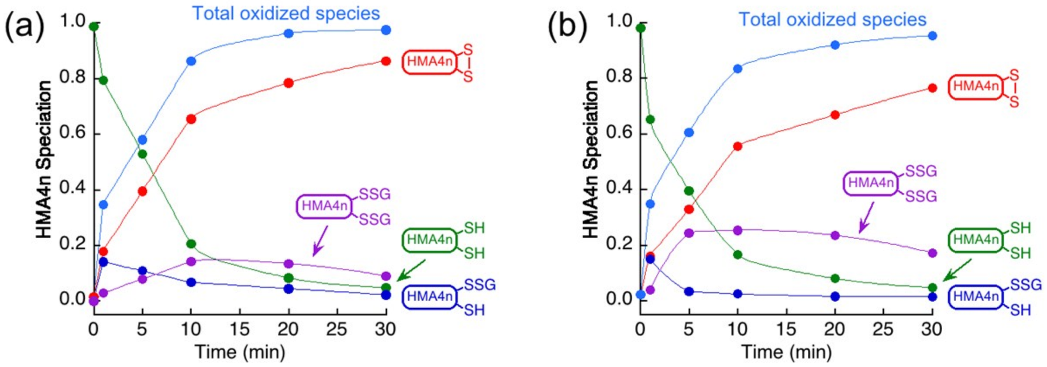

Figure S2. Protein speciation and reaction progress analysis upon oxidation of HMA4n(SH)$_2$ (10 μM) in deoxygenated KPi buffer (50 mM, pH 7.0) containing GSSG (400 μM)/GSH (40 μM): (a) with EcGrx1 (100 nM) as catalyst; (b) with EcGrx1-C14S (50 nM) as catalyst.

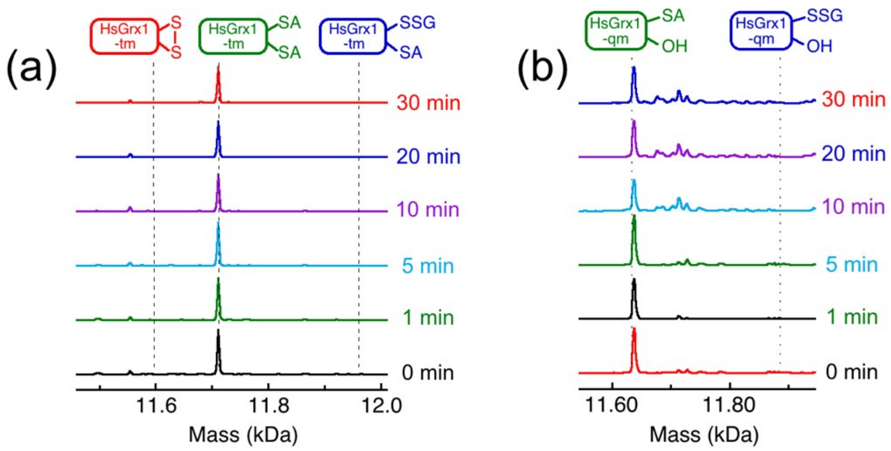

Figure S3. IAA/ESI-MS speciation analysis for HsGrx1-tm (a) and HsGrx1-qm (b) during the course of the catalytic reduction of HMA4n(SS) by GSH/Grx showed in Figure 4c.

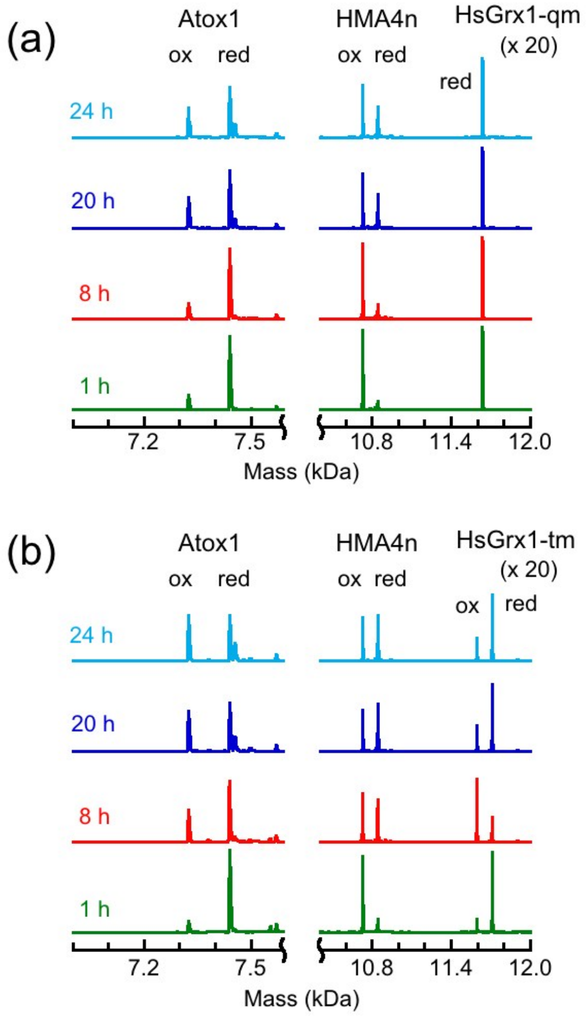

Figure S4. IAA/ESI-MS analysis of reaction progress and protein speciation for thiol-disulfide exchange between Atox1$(SH)_2$ and HMA4n(SS) (each 10 μM) in deoxygenated Mops buffer (50 mM, pH 7.0) with either monothiol or dithiol Grx enzymes:

(a) monothiol HsGrx1-qm (0.5 μM) (indistinguishable with no enzyme);

(b) dithiol HsGrx1-tm (0.5 μM).

References

(1) Ukuwela, A. A.; Bush, A. I.; Wedd, A. G.; Xiao, Z., Reduction Potentials of Protein Disulfides and Catalysis of Glutaredoxin1 Enzymes for Glutathionylation and Deglutathionylation, *Biochem. J.* **2017**, *474,* 3799-3815.

(2) Xiao, R.; Lundström-Ljung, J.; Holmgren, A.; Gilbert, H. F., Catalysis of Thiol/Disulfide Exchange: GLUTAREDOXIN 1 AND PROTEIN-DISULFIDE ISOMERASE USE DIFFERENT MECHANISMS TO ENHANCE OXIDASE AND REDUCTASE ACTIVITIES, *J. Biol. Chem.* **2005**, *280*, 21099-21106.

(3) Bushweller, J. H.; Aslund, F.; Wuthrich, K.; Holmgren, A., Structural and functional characterization of the mutant Escherichia coli glutaredoxin (C14--S) and its mixed disulfide with glutathione, *Biochemistry* **1992**, *31*, 9288-9293.

(4) Åslund, F.; Nordstrand, K.; Berndt, K. D.; Nikkola, M.; Bergman, T.; Ponstingl, H.; Jörnvall, H.; Otting, G.; Holmgren, A., Glutaredoxin-3 from *Escherichia coli*. Amino acid sequence, 1H and 15N NMR assignments, and structural analysis, *J. Biol. Chem.* **1996**, *271*, 6736-6745.

(5) Berardi, M. J.; Bushweller, J. H., Binding specificity and mechanistic insight into glutaredoxin-catalyzed protein disulfide reduction1, *J. Mol. Biol.* **1999**, *292*, 151-161.

(6) Lillig, C. H.; Prior, A.; Schwenn, J. D.; Aslund, F.; Ritz, D.; Vlamis-Gardikas, A.; Holmgren, A., New thioredoxins and glutaredoxins as electron donors of 3'-phosphoadenylylsulfate reductase, *J. Biol. Chem.* **1999**, *274*, 7695-7698.

(7) Berndt, C.; Schwenn, J.-D.; Lillig, C. H., The specificity of thioredoxins and glutaredoxins is determined by electrostatic and geometric complementarity, *Chem. Sci.* **2015**, *6*, 7049-7058.

(8) Åslund, F.; Ehn, B.; Miranda-Vizuete, A.; Pueyo, C.; Holmgren, A., Two Additional Glutaredoxins Exist in Escherichia coli: Glutaredoxin 3 is a Hydrogen Donor for Ribonucleotide Reductase in a Thioredoxin/Glutaredoxin 1 Double Mutant, *Proc. Natl. Acad. Sci. U.S.A.* **1994**, *91*, 9813-9817.

(9) Mieyal, J. J.; Starke, D. W.; Gravina, S. A.; Hocevar, B. A., Thiotransferase in human red blood cells: kinetics and equilibrium, *Biochemistry* **1991**, *30*, 8883-8891.

(10) Badarau, A.; Dennison, C., Copper trafficking mechanism of CXXC-containing domains: insight from the pH-dependence of their Cu(I) affinities, *J. Am. Chem. Soc.* **2011**, *133*, 2983-2988.
